# Macrophages on demand: How tissue trauma shapes their role

**DOI:** 10.1007/s00068-025-03050-y

**Published:** 2026-01-13

**Authors:** Lara Johnsen Stefani, Stefan Wohlgemuth, Lena Schütte, Rebecca Halbgebauer, Christian B. Bergmann, Markus Huber-Lang, Lisa Wohlgemuth

**Affiliations:** 1 Institute of Clinical and Experimental Trauma-Immunology, University Hospital Ulm, Ulm, Germany; 2 Department of Trauma, Hand, Plastic and Reconstructive Surgery, University Hospital Ulm, Ulm, Germany

**Keywords:** Macrophages, TBI, Brain-lung-axis, Liver, Intestine, Polytrauma, Immunomodulation

## Abstract

Macrophages, renowned for their plasticity, are central to both the immune response and tissue repair following physical trauma. This review delineates how tissue trauma dynamically modulates macrophage function across organs, highlighting their dichotomous roles in promoting inflammation versus regeneration. After injury, macrophages shift along a continuum from pro-inflammatory to pro-regenerative states, influenced by local and systemic cues, injury type, and microenvironmental factors, including damage-associated molecular patterns, and cytokines leading to pronounced organ-specific differences. The temporal and spatial dynamics of macrophage recruitment – from resident pools or via monocyte influx – dictate not only healing outcomes but also the risk of organ dysfunction and chronic inflammation. Emerging immunomodulatory strategies, encompassing stem cell therapies, pharmacological phenotype modulation, and microbiome-targeted approaches, underscore the clinical potential of precise macrophage-targeted interventions. Understanding macrophage adaptability post-trauma is crucial for devising organ- and context-specific therapies to optimize tissue repair and minimize adverse outcomes.

## Introduction

Macrophages play a pivotal role in both tissue destruction and tissue development. Thus, following severe tissue trauma, macrophages are crucial in debris clearance and the induction of regenerative processes. However, macrophages can also aggravate the vicious immunopathological circle after traumatic injury driving inflammation, auto-aggression, as well as organ dysfunction and multiple organ failure [1]. The word “macrophages” originates from the ancient Greek words “macro” (large) and “phagein” (eating). As a major function, their “eating capacity” helps clearing damaged tissue and dangerous invaders. Traditionally, macrophages are classified devided into two main phenotypes: M1 and M2. M1 macrophages are induced by microbial products and tumor necrosis factor alpha (TNF-α), functioning as pro-inflammatory cells that mediate immune defense. In contrast, M2 macrophages exhibit anti-inflammatory properties and can be further subdivided into several distinct subtypes. Among these, M2a macrophages are induced by interleukin-4 (IL-4) and interleukin-13 (IL-13) and play a pivotal role in wound healing. M2b and M2c macrophages are regarded as regulatory macrophages with potent anti-inflammatory activity. The former are induced by immune complexes and lipopolysaccharides (LPS), whereas the latter arise in response to interleukin-10 (IL-10) and glucocorticoids. M2d macrophages have been described as a distinct subtype induced by adenosine A2A receptor agonists, primarily exhibiting pro-angiogenic properties [2, 3]. Nevertheless, macrophages are highly plastic and adaptable in their functions. Several phenotypes have been defined by single cell [4] and artificial intelligence [5] approaches. After trauma, the temporo-spatial responses of macrophages not only depend on the severity of the impact but also on the coexistence of ischemia/reperfusion injury, hemorrhagic shock, or thermal injury [6]. Along the time axis of healing, a well-established paradigm proposes a trauma-induced polarization from the pro-inflammatory M1 phenotype towards the pro-regenerative M2 phenotype [7]. However, a recent investigation in a murine polytrauma model drew a more nuanced picture showing that the macrophage numbers and function differed between the different injured and remote organs. At a closer look, it seems that macrophages are recruited from remote uninjured sites to the injured problem zone, where they can switch to a pro-regenerative phenotype. For example, a reduction of the resident macrophage pool in the spleen and lung was observed early up to 7 days after trauma, whereas synchronically, an increase in macrophages in the directly injured liver was detected, including an M2-phenotype acutely after trauma [8]. On a subcellular level, other studies have focused on the mechanisms for an M1/M2 phenotype switch suggesting for example exposure to collagen-derived phosphatidylserine liposomes [9], extracellular vesicles [10] or even hypothermic conditions [11] as a driver of such polarization. Furthermore, extracellular matrix (ECM) from tissue debris differentially altered macrophage function: whereas brain ECM induced an M1-like phenotype, urinary bladder ECM biased macrophages towards an M2-like phenotype, mainly caused by the rich hyaluronic acid components [12].

However, although more than three decades ago, altered macrophage surface expression profiles and functions have been described after tissue trauma [13], the specificity and clinical consequences of these changes are still unclear. In surgical patients suffering from septic complications, enhanced numbers in circulating monocytes and altered surface expression profiles including a reduction of human leukocyte antigen (HLA)-DR expression have been described for circulating monocytes with suppressed INF-$\gamma$ synthesis being associated with sepsis severity and a poor outcome [14].

The diagnostically well-accessible circulating monocytes can rapidly augment the pool of resident macrophages of the corresponding organs. The macrophages in the various spatial regions, e.g. Kupffer cells in the liver, alveolar or pleural macrophages in the lungs, bone macrophages, red pulp macrophages in the spleen, or peritoneal macrophages [15], are less accessible and may exhibit diverse functions depending on the trauma impact, injury pattern and time elapsed after trauma.

Thus, this review focuses on how macrophages adapt their immuno-pathophysiological role within traumatic injuries across different organs and finally provides some exemplary immunomodulatory therapeutic approaches.

## Differential role of macrophages in traumatic brain injury

Traumatic brain injury (TBI) is a disorder that globally affects more than 25 million people every year with increasing incidence over the last decades [16]. TBI is characterized by the “primary injury”, which is limited to the physical injury inflicted by the trauma vector force and the subsequent initial minutes. The subsequent “secondary injury” is the prolonged endogenous immuno-pathophysiological response [1] that can extend from the first few hours to months, if not years. The latter is also associated with chronic cerebral hypoxia-ischemia, neurodegeneration, and demyelination, all of which are risk factors for impairment of motor and cognitive functions, development of neuropathologies (Alzheimer’s, Parkinson’s, amyotrophic lateral sclerosis), and mortality [17, 18]. The “secondary” TBI is driven by a robust inflammatory process in which the recruitment and polarization of the resident microglia macrophages as well as bone-marrow-derived monocytes that differentiate into macrophages play an important role. Given the plasticity of macrophages adjusting to the respective micromilieu, different markers and receptor proteins of macrophages may be associated with different effects on debris clearance and healing following trauma.

With the mobilization of macrophages near the trauma site, both M1- and M2-like phenotypes are expressed in a way that constitutes a heterogeneous gradient spectrum rather than a distinct form of one or the other [19–23]. This has been highlighted by a sophisticated approach using flow cytometry and RNA sequencing, emphasizing the need of detailed phenotypic identification with multiple chemoattractants and macrophage-specific markers [19]. However, since the gene expression signature and markers of both M1 and M2 may coexist in the same cell, functional analyses defining differences in plasticity ex vivo and in vivo may be necessary. Murine TBI macrophages exhibited a common behavior of early M1-like and M2-like responses, quickly substituted by an increasing accumulation of M1-like and mixed transitional (Mtran) phenotypes [20]. The Mtran subtype is characterized by high expression levels of NADPH oxidase 2 (NOX2), proinflammatory signaling and secretion of reactive oxygen species (ROS). These functions appear mostly in infiltrating macrophages, peaking around 7 days post injury. Of note, when inhibiting NOX2 with gp91ds-tat delayed treatment, the M2-like phenotype was favored while reducing oxidative stress in neurons a week post injury. There is also evidence that NOX2 deficiency may drive an anti-inflammatory response and result in an improved motor recovery and reduced neurodegeneration 21 days after brain contusion [23, 24]. The NOX2-dependent phenotype shift was not possible for bone-marrow-derived macrophages (BMDMs) upon exposure to exclusive pro- or anti-inflammatory stimuli. However, a combined stimulation by LPS and IL-4 finally resulted in cellular activation. NOX2^−/−^ BMDMs exhibited decreased levels of STAT1, but higher expression levels of STAT3 and the anti-inflammatory IL-10, which in consequence favors an M2-like phenotype in the cortex at the site of cerebral cortex injury [21, 25, 26].

An established marker for the M2-phenotype, CD163, has been shown to be expressed in rat macrophages in the meninges, choroid plexus, and perivascular space. However, upon TBI, CD163^+^ macrophages accumulate at the lesion site two days after injury and beyond [27]. Furthermore, these CD163^+^ macrophages co-expressed heme oxygenase-1 (HO-1) induced by hemoglobin complexes e.g., due to hemorrhage after trauma [27]. Functionally, activation of the HO-1 complex results in reduction of oxidative stress and in promoting anti-inflammatory effects. In contrast, pathogen-associated molecular patterns (PAMPs) including LPS and inflammatory mediators including IFN-𝛾 rather downregulated this HO-1 expression [27]. The removal of hemoglobin-haptoglobin complexes and TNF-related weak inducer of apoptosis (TWEAK) is part of the role of CD163 by subsequent endocytosis, clearance, and inflammatory resolution, suggesting a rather protective role after tissue trauma [27, 28].

Moreover, aging may influence the macrophage transcriptional signature after trauma, e.g. shifting it more towards a pro-inflammatory M1 phenotype [29]. In the case of TBI, an exacerbated pro-inflammatory cytokine profile including long-term MAPT/Tau pathologies and cognitive dysfunctions are often seen. hTau mice exposed to TBI exhibited an increase in macrophages and microglia at the ipsilateral hippocampus associated with a hyperactive response. This macrophage response led to a greater vulnerability to additional immunological challenges and to a delay in both motor and memory tasks [30].

## Response of macrophages in thorax trauma depends on their origin and M1/M2-balance

Thorax trauma with contusion of the lungs is occurring in 40% of polytraumatized patients and significantly enhances the morbidity and mortality by 14–40% depending on the severity [31]. Depending on the trauma impact, there is a differential role of pulmonary macrophages [6]. The main pulmonary macrophage population found in the lungs represents resident alveolar macrophages (AMs) which play a crucial role in the maintenance of lung homeostasis by immune surveillance and defense. Further macrophage subtypes are tissue-resident alveolar macrophages (TR-AMs), monocyte-derived alveolar macrophages (Mo-AMs), and pulmonary interstitial macrophages (PIMs) [32]. In steady state conditions, the AMs exert mainly immunosuppressive functions, and appear barely plastic when activated [32]. Mo-AMs are more flexible in their response to the corresponding milieu. However, they can activate and generate cytokine cascades and even drive anti-inflammatory mechanism to regain the immune balance following severe inflammation. PIMs take essential roles in immune regulation, type II inflammatory response, and pulmonary fibrosis [32].

Concerning the AM-driven acute inflammatory response after trauma, a murine blunt chest trauma model resulted in increased upregulation of MCP-1, MCP-2, and CINC-1 mRNA in AMs and revealed release of MCP-1 and MIP-2 proteins by AMs compared to sham mice [33, 34]. Monocytes and AMs also exhibited upregulated CCR2 mRNA levels which might be explained by the increase in CCR2high (inflammatory) monocytes migrating from the bone marrow to the lungs. Furthermore, lung trauma with corresponding exposure to damage-associated molecular patterns (DAMPs) and subsequent inflammation facilitates development of infections, clinically manifested as pneumonia, which in turn drives inflammation and thus a vicious circle escalating in development of organ failure [1]. On a molecular level, AMs are regulated by their micromilieu of DAMPs, PAMPs, and thrombo-inflammatory mediators as a trade-off between reactivity and tolerance [35]. AMs may turn “paralyzed” through inflammatory mediators, including signal-regulated protein a (SIRP$\alpha$) and exhibit an impaired phagocytic rate. In this context, antibody blockage of SIRPa receptor recovered phagocytotic function and may thus support the immune response and reduce pneumonia development [35].

The rapid inflammatory response driving posttraumatic acute respiratory distress syndrome (ARDS) is mainly driven by an early M1 activation associated with an exudative phase, pro-inflammatory cytokine storm and neutrophil recruitment in the lungs. The M1 phenotype is regulated, among others, by STAT1, SOCS1, SOCS3 and IRF-5. Following this first phase, M2 macrophages are responsible for resolution and recovery with anti-inflammatory cytokines, promoting healing mediated for example by STAT6, SOCS1, SOCS3 and IRF-4 [36, 37]. Concerning the proposed polarization of macrophages to an M2-like phenotype, the phagocytosis receptor Mer proto-oncogene tyrosine kinase (MerTK) on macrophages seem to play a crucial role. In a remote lung injury model caused by hind limb ischemia reperfusion injury, the MerTK-promoted tissue repair helped M2-like macrophages resolve inflammation [38, 39]. Furthermore, metalloproteinases (MMP), specifically ADAM17, which are activated after trauma, can cleave the MerTK-receptor which in turn result in an impairment and delay of inflammatory resolution [38]. Another mechanism of compromised inflammatory clearance is presented by a specific AM population anchored to the epithelium. These AMs were found to limit LPS-induced inflammation by inducing epithelial Ca^2+^ waves and subsequent activation of a pro-survival Akt kinase [40]. However, the disturbance of this interaction, e.g. by epithelial necrosis and neutrophil recruitment, contributes to the development of ARDS.

A shift in the M1/M2 phenotypic balance towards M1 macrophages (by reduction of M2 macrophages) has been proposed in the pathogenesis of ARDS following experimental hemorrhagic shock/resuscitation in mice [41]. The drawn BAL fluids treated ex vivo with LPS revealed characteristic pro-inflammatory responses reflected by enhanced NF-𝜅B translocation, higher TNF- and iNOS-RNA expression levels, and lower rates for IL-10. When catalase was inhibited, oxidative stress enhanced the LPS-induced inflammatory response and impaired IL-4 generation and in consequence the expression of Kruppel-like factor, all contributing to a decrease of the M2 phenotype. Similar alterations were found for hyperoxic conditions which reduced IL-4-induced macrophage polarization towards the M2-like phenotype [41]. The different macrophage phenotypes are also associated with different metabolic states. M1 macrophages exhibit enhanced rates in glycolysis, pentose phosphate pathway and fatty acid synthesis, and disturbed tricarboxylic acid (Krebs cycle). These metabolic changes help generating pro-inflammatory cytokines. In contrast, M2 macrophages are mainly driven by oxidative phosphorylation, glutamine metabolism and fatty acid oxidation which generate a considerable amount of ATP necessary for upregulation of genes coding for tissue repair. Additionally, the production of 𝛼-ketogluturate by glutamine hydrolysis contributes to alternative activation of macrophages [37].

Additional pathomechanistic drivers of early posttraumatic ARDS are necrosis and apoptosis of macrophages. In a mouse lung contusion model, the resultant hemorrhagic “flooding” of the alveolar space increased levels of IL-1ꞵ and IL-6 with a sustained local neutrophilic inflammation, while in contrast, the resident alveolar macrophages got lost early in the posttraumatic course due to necrosis/apoptosis. An early decrease in AM numbers has also been attributed to an auto-amplification loop of cell death and inflammation [42, 43]. In later phases after pulmonary trauma, macrophages seem also crucially involved. For example, during the clearance of damaged cells and repair phase following traumatic lung injury, IL-4 and IL-13 polarize macrophages shift to M2-like phenotype, which can generate TGF-ꞵ1 promoting fibroblast proliferation and differentiation. These changes eventually lead to fibrosis, representing a major clinical problem post lung trauma [44].

Of note, age also appears to play a major role in AM responses following traumatic injury [45]. Elder mice exhibited higher expressions of mrc1, chil3 and mfge8, all of which led to an M2-like phenotype. The decrease in tlr2 and ilb1 characterize deficiencies in TLR2 and inflammasome signaling, leading to failure of clearing bacteria. After exposure to a peripheral (scald) trauma, the transcriptional response of AMs from aged mice was overall impaired with a dysregulated corticosteroid response [45]. Vice versa, a senescent environment reduced the capacity of AMs to take up and clear senescent fibroblasts or epithelial cells and even bystander corpse, via a disturbance of the SIRPα-CD47-SHP-1 axis [46]. However, further research is required to define to what extent senescence processes in concert with chronic inflammation drive direct lung trauma and fibrotic processes.

## Role of macrophages in the liver and intestine after abdominal trauma

Abdominal trauma in severely multiple injured patients commonly involves blunt hepatic trauma, which is the second most injured organ next to the spleen. Many research groups have highlighted the liver´s vulnerability in trauma [47–49] likely associated due to its size and its anterior location in the abdomen [50]. The main complication of severe liver injury following blunt abdominal trauma is an uncontrolled bleeding in consequence of lesions of the parenchyma including hepatic veins [50].

As the central metabolic and most regenerative organ, the liver encounters tissue-resident macrophages that are predominant in the healthy naïve liver and represent the key phagocytes [51]. Those constitutively resident macrophages are the so-called Kupffer cells that were first discovered in 1876 by Karl Wilhelm von Kupffer [52]. Kupffer cells (KCs) are indispensable in maintaining tissue and systemic homeostasis by phagocytosis of exogenous (e.g., bacteria) but also endogenous (e.g., aged erythrocytes) particles. They belong to the “first line of defense” against bacteria due to their location in the liver sinusoids processing blood from the gastrointestinal tract through the portal vein [53, 54]. In many diseases and after hepatic injury Kupffer cells (KCs) exert a key role in mounting an immunological response [55]. KCs get activated via pattern recognition receptors (PRRs) that are detecting DAMPs, such as High Mobility Group Box 1 (HMGB1) or mitochondrial DNA, and can trigger and aggravate the inflammatory response [56]. Activated KCs provide signals to regulate the hepatic response by secreting various proinflammatory mediators including cytokines (e.g., TNF, IL-1β and IL-6) and chemokines (e.g., CCL2 and CCL5) [15, 57]. After abdominal trauma, KCs are one of the earliest and most potent sources of actively secreted HMGB1 due to the liver’s central immune surveillance role [49]. With that, KCs boost the inflammatory response and recruit, among others, hepatic monocyte-derived macrophages (MoMΦs) from the blood stream. Experimental direct mechanical trauma of the liver resulted in hepatic hypermetabolism associated with increased intracellular calcium concentration and prostaglandin E_2_ (PGE_2_) production of KCs [58]. Upon liver injury, MoMΦs are extensively recruited through the binding of MCP-1 (also known as the chemokine ligand 2, CCL2) secreted by KCs and the corresponding chemokine receptor CCR2 on circulating monocytes [59]. After liver injury, immigrated MoMΦs are part of the repair processes including phagocytosis of dead cells (linked with excessive ROS generation) and promotion of hepatic tissue healing [60]. However, excessive MoMΦs activation may also worsen the liver damage through prolonged inflammation [51]. After acute liver injury, KCs produce anti-inflammatory mediators like IL-1 receptor antagonist (IL-1Ra) as well as hepatocyte growth factor (HGF) and vascular endothelial growth factor (VGEF) for tissue remodeling [57]. Many experimental liver trauma studies combine abdominal trauma and hemorrhage to analyze macrophage pathologies as this combination is present in 20% of patients with severe abdominal injuries [61, 62]. Following trauma-hemorrhage, activation of KCs represent a main source of MCP-1 production which in turn plays a major role in development of remote organ dysfunction [63]. Nevertheless, pathophysiological changes of liver macrophages beyond the described M1/M2 phenotype switch remain unclear in the context of abdominal trauma and warrants further trauma-specific investigations.

Abdominal trauma may also injure hollow organs like the intestine [64, 65]. The intestine is a rather vulnerable organ as it requires a certain degree of permeability to absorb nutrients and fluids [66]. This unique morphology creates an easy entry gate for damage molecules and pathogens and requires a unique and potent immune system. Besides other leukocytes, intestinal macrophages make up the largest and most dense population of resident as well as transient mononuclear phagocytes in the intestine during homeostasis and therefore represent key players in the clearance of apoptotic cells and cellular debris [67]. Intestinal macrophages exhibit unique characteristics that differ from other macrophage populations as they possess some of the hallmarks of both M1 and M2 macrophages. They express high levels of MHC class 2 receptors like M1 macrophages and also produce anti-inflammatory mediators like M2 macrophages (e.g., IL-10) [68]. The continuous release of TGF-β and IL-8 by mucosal epithelial cells drives a steady recruitment of monocytes from the peripheral blood [69, 70]. Under the influence of TGF-β, IL-10, M-CSF, CX3CL1, and CCR2, these monocytes differentiate into characteristic intestinal macrophages [71, 72]. Due to significantly altered receptor profiles including reduced expression of Toll-like receptors (TLRs) and Fc gamma receptors as well as active inhibition of pro-inflammatory signaling pathways and high IL-10 production, intestinal macrophages exhibit a pronounced state of immunological tolerance [69, 72, 73]. Despite their strong phagocytic capacity, these macrophages do not trigger inflammatory responses [73, 74].

However, in the context of severe abdominal trauma, macrophages lose their immunological tolerance, reflecting once more their high functional plasticity [15, 75]. The release of DAMPs and increased exposure to bacterial products following the breakdown of the intestinal barrier, result in a strong activation of pro-inflammatory signaling pathways (e.g., NF-𝜅B, MAPK) via corresponding PRRs [1, 76–78]. This leads to a phenotypic shift toward an M1-type macrophage profile, marked by elevated production of pro-inflammatory cytokines such as IL-1β, TNF, and IL-6 [8, 73, 77]. Furthermore, new monocytes are recruited, which differentiate into proinflammatory macrophages and partially replace the existing resident macrophage population [1, 77]. This proinflammatory milieu is sustained by the breakdown of natural humoral tolerance, exemplified by the diminished continuous production of TGF-β and IL-10 [76–79]. The altered macrophage biology not only contributes to the local or systemic inflammatory response but also furthermore created a neurodegenerative environment in the gut followed by subsequent motility disturbances [80].

Special attention should be given to the gut microbiome as an integral component of the intestine. Following trauma, the gut microbiome undergoes significant changes from a physiological balance toward an overgrowth of more pathogenic bacteria, such as *Enterobacteriaceae* and *Clostridium* species [81]. This dysbiosis promotes a phenotypic shift in intestinal macrophages toward a highly pro-inflammatory state. The combination of these two factors further increases intestinal permeability, facilitates the translocation of pathogenic microorganisms, and contributes to sustained severe inflammation. In turn, this can lead to downstream complications including multiple organ failure [68, 82]. Underlying mechanistic consequences of the cross-talking microbiome and immune system for the intestinal macrophages need further studies which may eventually reveal novel avenues for therapeutic modulation.

## Role of macrophages in the context of polytrauma

Systemic changes in macrophage behavior in polytrauma revealed distinct response patterns in a dynamic manner in both animal and human studies. Macrophages in trauma consist of circulating blood monocytes (Ly6C^hi^CCR2^+^ in mice and CD14 ^++^ CD16^-^ in humans) that are systemically released post tissue injury and then transmigrate to the injury site and differentiate into macrophages [83]. In addition, activated resident macrophages promote monocyte extravasation to the injury site, where they differentiate into macrophages and expand the local population. Neutrophils further aid macrophage recruitment by upregulating MCP-1 and CCL3. Within the tissue, macrophages secrete factors like IL-1β, TNF, IL-6, and CCL2 to recruit fibroblasts, mesenchymal stem cells (MSCs), and progenitor cells, functioning in a pro-inflammatory role in the early stage of wound healing [84–86] and later converse into an M2-like reparative phenotype [87].

In a murine polytrauma model, it was shown that trauma induction triggers a biphasic macrophage response. The initial inflammatory phase is characterized by increased infiltration of CD68⁺ and inducible nitric oxide synthase⁺ (iNOS⁺) M1 macrophages, accompanied by elevated inflammatory markers [8]. This is followed by a compensatory anti-inflammatory phase marked by a shift toward Arginase 1⁺ M2 macrophages, potentially facilitating tissue repair [8]. Human studies have demonstrated similar patterns through systemic inflammatory response syndrome (SIRS) and compensatory anti-inflammatory response syndrome (CARS) [88]. Importantly, tissue-specific analyses in mouse models have revealed that macrophage responses vary significantly across different organs [89]. In physiological wound healing, there is an M1 macrophages transition to an alternatively activated (M2) phenotype, marked by secretion of IL-10, TGF-β, and expression of Arginase-1 [90]. However, in polytraumatized patients, this M1/M2 transition is often impaired or delayed, resulting in prolonged inflammation, impaired tissue repair, and an increased risk of chronic wound development and fibrosis. Several animal studies revealed a dependency of timely wound healing on physiologic macrophage function [91, 92].

A recent study investigated the role of autocrine TGF-β1 signaling in macrophages during aberrant wound healing leading to heterotopic ossification (HO) [93]. Using a murine model, they demonstrated that TGF-β1 signaling within macrophages, rather than mesenchymal stem/progenitor cells, is pivotal in HO formation. Single-cell transcriptomics revealed increased TGF-β1 activity in early infiltrating macrophages. Of note, genetic deletion of TGF-β receptor type 1 (Tgfbr1/Alk5) in these cells led to exacerbated HO, whereas systemic administration of a TGF-β1/3 ligand trap (TGF-βRII-Fc) reduced HO formation and delayed macrophage infiltration [93]. The study underlines the potential of macrophages to direct other cells, likely fibroblasts to physiologically control tissue healing which may reflect a potential therapeutic target.

Polytraumatized patients often exhibit soft tissue injury and especially muscle injury, measured by systemic myoglobin release, which was also shown to positively correlate with the injury severity score (ISS) [94]. Muscle laceration resulted in accumulation of macrophages in murine skeletal muscle for at least 21 days, concurrent with limited myofiber regeneration and persistent collagen deposition [95]. In this context of wound healing, the M2a macrophage subtype in general plays a distinct role by producing pro-fibrotic factors [96]. Of note, murine muscle macrophages do not display classical M1 or M2a phenotypes. Instead, early after injury, macrophages upregulated gene expression profiles associated with both M1 and M2a activation, followed by a subsequent downregulation of most assessed markers. IL-10 mRNA and protein levels are specifically and markedly elevated in macrophages isolated from muscle at 3 days post-injury. Moreover, flow cytometry identified macrophage subpopulations distinguished by high or low TNF expression, which overall did not correspond distinctly to canonical M1 or M2a phenotypes [95]. Another murine study investigated muscle damage and asked if local cryotherapy reduces it. The authors found cryotherapy after closed soft tissue injury reduces macrophage invasion, restored diminished functional capillary density, decreased intramuscular pressure, and attenuated tissue damage [97]. These findings suggest that the impaired macrophage M1/M2 shift observed in other tissues, such as skin [87], might not be found in muscle tissue, but inflamed macrophages rather detrimentally affect tissue healing. A specific future question to be addressed is the role of macrophages in skeletal muscle, given the large number of cells that are injured, which might trigger adverse innate immune cell activation.

## Immunomodulation of macrophages – beneficial for organ performance post trauma?

In general, following trauma, an early M1 response is observed within the first hours to days. Subsequently, the immune response transitions towards an anti-inflammatory M2 profile [8, 98]. The precise course of this process is determined by the surrounding tissue and the specific pattern of injury. Herein, as outlined in the preceding sections, the post-traumatic immune reaction does not represent a purely M1 or M2 response, but rather a continuum characterized by concurrent pro- and anti-inflammatory activity, as well as the involvement of tissue-specific cell populations [8, 98, 99]. Immunomodulation of macrophages post trauma might either aim to attenuate the pro-inflammatory M1-like phenotype or to enhance the regenerative repair M2-like phenotype. Some of these approaches within the specific tissue/organ will be addressed in the following sections.

## Modulation of macrophages after TBI

Macrophages significantly contribute to the immunopathophysiology of TBI [1]. Following TBI, resident microglial cells exhibit a heterogeneous response. On the one hand, they generally adhere to the established paradigm of an initial M1 activation during the first hours followed by a delayed M2 response. However, they also display a mixed phenotype already in the immediate aftermath of the insult [100, 101]. From a temporal perspective, it is possible to intervene in the inflammatory process at a very early stage, within the first 24 h. In a murine cortical contusion TBI-model, intracerebral human neural stem cells (hNSC) transplants with stem cells not only decreased abnormal amyloid precursor protein (APP) accumulation and axonal injury but also modulated microglia/macrophages towards an M1/M2-phentoypic transition. Mechanistically, the M1/M2-switch induced in the brain an IL-4Ra signature and reduced IFN-𝛾Rꞵ levels, resulting in an anti-inflammatory response with potential neuroprotective features [21]. Mesenchymal-mediated polarization can also occur through modulation of CX3CR1 receptors by releasing CX3CL1, expressing IL-1 receptor antagonist, diminishing NF𝜅B, and secreting PGE_2_ and anti-inflammatory proteins [102, 103]. To influence the M1/M2 shift of microglia after TBI it was further shown that inhibition of HMGB1 protein by glycyrrhizin favors the M2 phenotype resulting in neuroprotective effects following TBI [104]. Furthermore, in rat spinal cord injury, an HO-1 overexpression led to an inhibition of Nod-like receptor protein 1 (NLRP1) protecting neurons from neuronal death [105]. For example, effective immune-engineering strategies include multipotent adult progenitor cells and umbilical cord cells transfected with synthetic IL-4 mRNA to induce stronger M2-like macrophages [26]. However, the exact effects on circulatory and resident macrophages in the context of TBI still need further evaluation.

### Thorax trauma: Therapeutic approaches based on macrophage polarization

Thorax trauma is frequently accompanied by complications previously mentioned. Thus, it is tempting to speculate that strategies to modulate the immuno-pathophysiology can balance trauma-caused inflammation including the transcriptional and translational modifications and finally improve the posttraumatic course.

Given the critical involvement of different types of AMs, various macrophage targeting strategies can be applied. Herein, the role of Mo-AMs in infection-induced lung injury and pulmonary fibrosis was emphasized [106]. Blockade of the CCL2–CCR2 axis limits the recruitment of Mo-AMs and targeting this pathway can ameliorate pulmonary fibrosis [106–108]. Experimental studies have demonstrated that naturally occurring compounds exert immunomodulatory effects on pulmonary macrophages. Smiglaside A activates the AMPK and PPARγ signaling pathways, thereby promoting a phenotypic shift toward M2 macrophages and attenuating acute lung injury [109]. Conversely, pro-inflammatory signaling can be for example suppressed through inhibition of the p38 MAPK/MK2 pathway by administration of dehydrocostus lactone [110]. It was shown that the flavonoid didymin as a compound that strengthens fatty acid oxidation results in the M1 to M2 phenotypic switch [37]. In addition to signaling and metabolic pathways, humoral markers also influence the macrophage-mediated pulmonary immune response. In ARDS, elevated levels of soluble PD-L1 are associated with a reduced number of Mo-AMs, which in turn correlates with improved clinical outcome [111]. Moreover, murine studies have identified SIRPα as a relevant biomarker and a potential therapeutic target. The downregulation of SIRPα is thereby linked to enhanced phagocytic activity and a reduction in pulmonary tissue damage [112]. The aforementioned therapeutic approaches generally involve early intervention in the inflammatory cascade, typically within the first hours following trauma. Suppression of the initial pro-inflammatory macrophage response or inhibition of monocyte–macrophage infiltration leads to an enhanced neutrophil-driven immune reaction, thereby exacerbating pulmonary injury [33, 43, 108]. It is tempting to speculate that some of the M1/M2-shifting immunomodulatory approaches may be successfully transferred to the trauma setting.

## Modulation of macrophages after abdominal injury

As liver damage is mainly studied in the context of acute or chronic injury by alcohol and hepatotoxic drugs, therapeutic approaches in the sense of liver damage after abdominal trauma is rare. Nevertheless, targeting the hepatic macrophages may represent a promising approach implicating inhibition of macrophage recruitment, activation and phenotype switch [113]. As the excessive recruitment of monocyte-derived macrophages in line with excessive macrophage activation is also present after blunt abdominal trauma, those therapeutic aspects are worth further investigation. As mentioned in the previous section, the CCL2-CCR2 binding plays a vital role in macrophage recruitment and therefore the CCR2 inhibitor propagermanium [114] as well as the CCL2 antagonist mNOX-E36 [115] may have therapeutic potential. As mentioned, MCP-1 secreted by KCs plays a crucial role in remote organ dysfunction. In this context, it was shown that administration of 17β-estradiol (E2) modulates MCP-1 release and reduces the detrimental effects [63]. After initial liver damage, the M1 phenotype including excessively recruited MoMΦs may at best switch to a regenerative M2 phenotype. Therefore, therapeutic considerations exist that directly address this phenotype change. For example, utilizing drug delivery systems in the sense of nanoparticles coated with e.g. small interfering RNA (siRNA) conjugated to mannose-modified trimethyl chitosan-cysteine (MTC), may specifically transport siRNA to macrophages via binding their mannose receptor (CD206) finally preventing excessive release of cytokines like TNF [116]. The impact of these bioengineering approaches and others [117] on liver macrophages in the setting of blunt abdominal trauma still needs further research.

Direct immunomodulatory options in the context of abdominal trauma are rarely addressed. There are general approaches among others focusing on the stimulation/regulation of the IL-10 production and therefore dampening systemic inflammation and inducing regeneration. A promising therapeutic approach for intestinal damage is to focus on the modulation of the microbiome. Herein, it was shown that probiotics modulate innate immune cells like macrophages [118]. For the probiotic species *lactobacillus casei* strain an modulatory impact on pro-inflammatory cytokines like TNF, IL-1β, IL-6 and IL-8 linked to the M1-like phenotype was emphasized [119–121]. But also butyrate, a major metabolite of intestinal bacteria showed an influence on cytokine production like IL-10 and IL-12 more linked to the regenerative phenotype [122]. Consequently, probiotics are also modulating key pro-inflammatory signal pathways like NF-𝜅B and MAPK [119–121]. Moreover, it was shown that depletion of intestinal macrophages by CSF-1R antibody treatment led to decreased neuronal death within the enteric neuronal system after surgical trauma [80]. A possible approach for tissue regeneration of the intestine after abdominal trauma was shown within the influence of WNT signaling pathways. Macrophages are in general the most potent source of WNT ligands. In this context, the association between increased WNT ligand expression and tissue regeneration was emphasized in a murine study of intestinal injury [123]. In temporal terms, abdominal trauma elicits a macrophage response that occurs with a similarly rapid onset as observed in other organ systems [8, 124]. The optimal timing for therapeutic intervention is reported heterogeneously across the available literature. Immunomodulatory effects of the gut microbiome have been demonstrated in in vitro co-culture experiments [119–121]. In animal models, a similar pattern is observed in thoracic trauma emerges. Very early intervention amplifies the inflammatory response and leads to increased intra-abdominal organ injury [115, 125]. An early, yet slightly delayed, modulation of the immune response – such as exemplified by the administration of 17β-estradiol (E2) – has been shown to beneficially attenuate the pro-inflammatory immune reaction and reduce end-organ damage [63]. However, immunomodulatory interventions regarding intestinal macrophages after abdominal trauma are currently underexplored despite a theoretically great potential.

## Conclusion

**Fig. 1:**
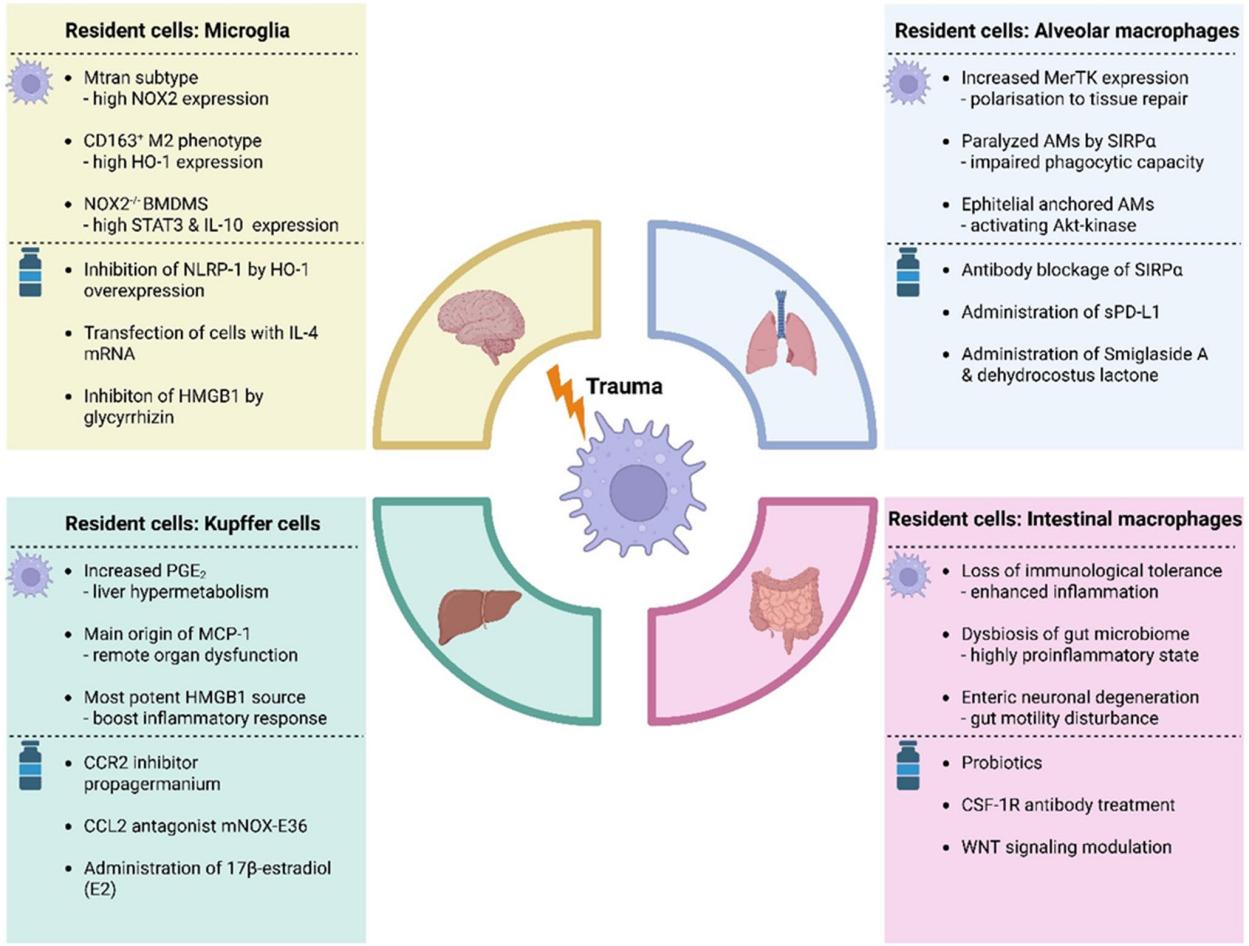
Organ-specific functional alterations of macrophages beyond the M1/M2 phenotype and exemplary immunomodulatory therapeutic approaches

In conclusion, after trauma, the phenotype-function-relation of macrophages considerably varies depending on the affected organ(s) and time elapsed after injury. Furthermore, different phenotypes may coexist depending on the functional demand to sense, capture, and clear damaged cells as well as induce, process, and complete tissue repair (Table 1). Overall, the highly plastic macrophages appear to function as adaptable “healers” and are capable to differently respond to trauma in a spatial and time dependent manner. Thus, any potential therapeutic interventions addressing macrophages require a highly targeted approach, which calls for both, novel immune monitoring and modulating principles (Fig. 1).

**Table 1.**
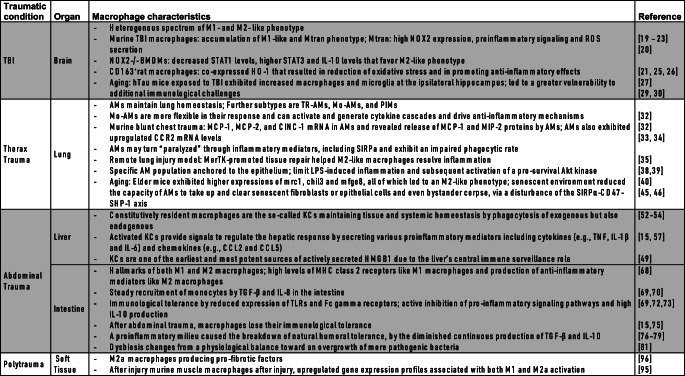
Summary of the organ-specific macrophage characteristics

Frequently injured organs including their resident macrophage population and organ specific alterations of resident but also other macrophage subtypes. Potential exemplary therapeutic approaches that target organ-specific macrophage plasticity and aim to modulate inflammation and enhance regeneration linked to macrophage pathophysiology. Created with biorender.com.

### Data availability and literature search

No datasets were generated or analyzed during the current study. The literature search for this narrative review was conducted by a broad initial screening using the multidisciplinary database PubMed open and searches in Google Scholar by L.J.S, L.S. and R.H and L.W. Herein, the terms for searching were mainly “macrophages” and “physical trauma”, used predominantly in combination, irrespective of the date of publication. Following this initial screening, specific organs relevant to the topic were identified, and a more detailed search was done including the organs by the authors that wrote the final manuscript including L.J.S., S.W., C.B.B., M.H.L, and L.W. In this stage, particular attention was given to interventional strategies targeting macrophages within the selected organs. Throughout the manuscript, original research articles and review articles were included, while especially studies addressing inflammatory conditions unrelated to physical trauma were excluded.

## Data Availability

No datasets were generated or analyzed during the current study. The literature search for this narrative review was conducted by a broad initial screening using the multidisciplinary database PubMed open and searches in Google Scholar by L.J.S, L.S. and R.H and L.W. Herein, the terms for searching were mainly “macrophages” and “physical trauma”, used predominantly in combination, irrespective of the date of publication. Following this initial screening, specific organs relevant to the topic were identified, and a more detailed search was done including the organs by the authors that wrote the final manuscript including L.J.S., S.W., C.B.B., M.H.L, and L.W. In this stage, particular attention was given to interventional strategies targeting macrophages within the selected organs. Throughout the manuscript, original research articles and review articles were included, while especially studies addressing inflammatory conditions unrelated to physical trauma were excluded.
